# Supplementary material for: Heart disease severity level identification system on Hyperledger consortium network
Source: PeerJ Comput Sci. 2023 Oct 12;9:e1626. doi: 10.7717/peerj-cs.1626 (PMC10588697; doi:10.7717/peerj-cs.1626)
Supplement: Supplemental Information 3 — The overall Hyperledger network implementation information is available in the test-network folder. The chaincode files, which are used for EHR data storage and retrieval, attribute-based access control policy implementation, and heart disease prediction with disease severity identification function, are contained in the medical-asset-transfer folder. [file peerj-cs-09-1626-s003.zip › Source-code/front_end/ehr/public/index.html]

React App

You need to enable JavaScript to run this app.
